# Association of Periodontal Pathogens and Their Inflammatory Mediators With Alzheimer’s Disease Neurodegeneration: A Systematic Review

**DOI:** 10.7759/cureus.104127

**Published:** 2026-02-23

**Authors:** Patrícia Arriaga, Kátia Vianna, Caroline Montez, Beatriz Panariello, Steve Tran, Diogo Rodrigues, Eliane Porto Barboza

**Affiliations:** 1 Hospital Dentistry, Multidisciplinary Center for Hospital and Intensive Dentistry, Rio de Janeiro, BRA; 2 Temporomandibular Disorders and Orofacial Pain, Hospital da Boca – General Hospital, Santa Casa da Misericórdia, Rio de Janeiro, BRA; 3 School of Dentistry, Augusto Motta University Centre (UNISUAM), Rio de Janeiro, BRA; 4 School of Dental Medicine, Lake Erie College of Osteopathic Medicine, Bradenton, USA; 5 Periodontics, National Institute of Dental Sciences (INCO 25), Niterói, BRA

**Keywords:** alzheimer’s disease, dementia, neurodegeneration, periodontal pathogens, periodontitis

## Abstract

Periodontitis is implicated in a range of systemic conditions, including cardiovascular disease, diabetes, and respiratory disorders. Emerging evidence suggests a link between periodontal infection, inflammation, and the neurodegenerative process of Alzheimer’s disease (AD). This paper aimed to systematically review observational studies examining the association of periodontal pathogens and their inflammatory products with AD neurodegeneration. The review was registered in the International Prospective Register of Systematic Reviews (PROSPERO - No. CRD42020150043). Methods followed the Preferred Reporting Items for Systematic Reviews and Meta-Analyses (PRISMA) guidelines. An electronic search (PubMed/Medical Literature Analysis and Retrieval System Online (MEDLINE), Web of Science, Scopus, Cochrane Library, grey literature) was conducted until September 2025 with no language or date restrictions. Two independent reviewers screened and extracted data. The risk of bias was assessed via the Risk Of Bias In Non-randomized Studies - of Exposures (ROBINS‑E) tool. Of 1,421 identified citations, eight studies met the inclusion criteria. Participant numbers ranged from 349 to 2,191, and ages ranged from 40 to 90 years old. Meta‑analysis was not feasible due to methodological heterogeneity. Risk of bias was moderate in five studies and serious in three. Findings indicated that higher serum IgG antibodies to periodontal pathogens and elevated inflammatory mediators, notably tumor necrosis factor-alpha (TNF‑α), correlated with greater cognitive decline and markers of AD neurodegeneration, including MRI outcomes and APOE ε4 status.

In conclusion, the current body of evidence suggests a potential association between periodontitis‑related inflammatory mediators, particularly TNF‑α, and elevated antibody responses to periodontal pathogens with AD progression. However, causality remains unestablished. Future prospective cohort and interventional studies are warranted to clarify the role of periodontal infection and inflammation in AD and to guide clinical strategies that may improve outcomes in AD populations.

## Introduction and background

Alzheimer’s disease (AD) is a neurodegenerative disorder that affects the central nervous system (CNS). It is characterized by gradual memory, learning, and orientation loss and results in progressive impairment and disability [1]. It is the most common form of dementia in older adults, and it is estimated that by 2050, one in every 85 individuals worldwide will be diagnosed with AD [2].

Although the precise etiology and pathophysiological mechanisms of AD remain unclear, inflammation is recognized as a key factor in its pathogenesis [1]. Evidence suggests that peripheral infections, vascular injury, and oxidative stress may exacerbate neuroinflammation and accelerate disease progression [3]. Systemic conditions, such as diabetes mellitus, cardiovascular disease, renal disease, and periodontal disease, have also been shown to influence the inflammatory state of the CNS [1,2].

Periodontitis is a chronic inflammatory disease of infectious origin, associated with Gram-negative anaerobic bacteria, which can trigger both localized and systemic infections in the host. Periodontal pathogens and their inflammatory products increase systemic inflammation and could accelerate the pathogenesis of AD, as endotoxins from periodontal bacteria, such as *Porphyromonas gingivalis,* *Tannerella forsythia*, *Treponema denticola, *and* Aggregatibacter actinomycetemcomitans, *can cross the blood-brain barrier and potentially accelerate the neurodegenerative processes characteristic of AD [4].

Proinflammatory cytokines, including interleukin (IL)-1β, IL-6, and tumor necrosis factor-alpha (TNF-α), released from inflamed periodontal tissues, may also enter the bloodstream or reach the brain through the trigeminal nerve. This can trigger or amplify neuroinflammation, contributing to the onset and progression of AD [5]. Therefore, this systematic review aimed to evaluate if there is an association between periodontal pathogens and their inflammatory products with the neurodegenerative mechanisms involved in AD.

## Review

Methods

This systematic review was registered in the International Prospective Register of Systematic Reviews (PROSPERO - No. CRD42020150043). The methodology used in this review followed the recommendations of the Cochrane Handbook for Systematic Reviews of Interventions [6]. The Preferred Reporting Items for Systematic Reviews and Meta-Analyses (PRISMA) checklist was followed to enhance the quality and transparency of the research. The clinical questions were broken down and organized using the Population, Exposure, and Outcomes (PEOS) strategy.

Main Question

Are periodontal pathogens and their inflammatory products associated with the neurodegenerative process of AD?

Clinical Relevance

Periodontitis can influence several systemic conditions, mainly including cardiovascular diseases, diabetes mellitus, premature low birth weight babies, and respiratory tract disorders [7]. However, several studies show a possible relationship between periodontitis and AD. Thus, this study aimed to identify scientific data on the influence of periodontal pathogens and their inflammatory products on the progression of AD.

Identification Strategy and Study Selection

An electronic search without date or language restriction was conducted in PubMed/Medical Literature Analysis and Retrieval System Online (MEDLINE), Web of Science, Scopus, and Cochrane Library until September 2025. In addition, grey literature was consulted (http://www.opengrey.eu) to identify new studies. The Medical Subject Headings (MeSH) terms used were “Alzheimer's disease,” “Dementia,” “Periodontal disease,” and “Periodontitis.” MeSH synonyms, related terms, and free terms were also included. Keywords were selected from the Health Science Descriptors (DECS) and MeSH of the US National Library of Medicine. The terms were combined to refine survey results. The combination of these descriptors is shown in Table 1.

**Table 1:** Electronic database used and search strategy.

Database	Search Strategy	
PubMed	#1 (((((Alzheimer’s disease [MeSH Terms]) OR Alzheimer’s disease [Title/Abstract]) OR dementia [MeSH Terms]) OR dementia [Title/Abstract]) OR cognitive impairment [Title/Abstract]) OR cognitive decline [Title/Abstract] #2 (((periodontal disease [MeSH Terms]) OR periodontal disease [Title/Abstract]) OR periodontitis [MeSH Terms]) OR periodontitis [Title/Abstract] #1 and #2 (((((((Alzheimer’s disease [MeSH Terms]) OR Alzheimer’s disease [Title/Abstract]) OR dementia [MeSH Terms]) OR dementia [Title/Abstract]) OR cognitive impairment [Title/Abstract]) OR cognitive decline [Title/Abstract])) AND ((((periodontal disease [MeSH Terms]) OR periodontal disease [Title/Abstract]) OR periodontitis [MeSH Terms]) OR periodontitis [Title/Abstract]))	
Scopus	#1 (TITLE-ABS-KEY ("Alzheimer's disease") OR TITLE-ABS-KEY (dementia) OR TITLE-ABS-KEY ("Cognitive Impairment") OR TITLE-ABS-KEY ("Cognitive Decline")) #2 (TITLE-ABS-KEY ("periodontal disease") OR TITLE-ABS-KEY (periodontitis)) #1 and #2 ((TITLE-ABS-KEY ("Alzheimer's disease") OR TITLE-ABS-KEY (dementia) OR TITLE-ABS-KEY ("Cognitive Impairment") OR TITLE-ABS-KEY ("Cognitive Decline"))) AND ((TITLE-ABS-KEY ("periodontal disease") OR TITLE-ABS-KEY (periodontitis)))	
Web of Science	#1 TOPIC: (Alzheimer’s disease) OR TOPIC: (dementia) OR TOPIC: (Cognitive Impairment) OR TOPIC: (Cognitive decline) Indexes=SCI-EXPANDED, SSCI, A&HCI, CPCI-S, CPCI-SSH, ESCI #2 TOPIC: (periodontal disease) OR TOPIC: (periodontitis) Indexes=SCI-EXPANDED, SSCI, A&HCI, CPCI-S, CPCI-SSH, ESCI #1 and #2	
Cochrane Library	"Alzheimer’s disease" OR dementia OR Cognitive Impairment OR Cognitive decline in Title Abstract Keyword AND periodontal disease OR periodontitis in Title Abstract Keyword (Word variations have been searched)	

This review included observational studies (cross-sectional, case-control, and cohort) evaluating the influence of periodontal pathogens and/or their inflammatory products on the progression of AD. Duplicate articles, literature reviews, records outside the proposed theme, case reports, in vitro studies, dissertations, theses, monographs, and animal studies were excluded.

The inclusion criteria were used to evaluate the design of the studies according to the PEOS as follows: Population (P): patients with AD; Exposure (E): IgG antibodies for periodontal pathogens and/or inflammatory products; Outcome (O): AD progression; Study design (S): observational studies (cross-sectional, case-control, or cohort).

The exclusion criteria for this review comprised studies that did not align with the PEOS framework or the objectives of the research question. Articles were excluded if they were not observational in design, including experimental studies, in vitro studies, animal studies, case reports, case series, literature reviews, meta-analyses, dissertations, theses, monographs, and conference abstracts. Studies were also excluded if they did not involve patients with AD, did not evaluate periodontal pathogens, IgG antibodies, or relevant inflammatory products, or did not assess outcomes related to AD progression. Records outside the scope of the proposed theme and duplicate publications were likewise removed.

Two independent reviewers (PA and CM) conducted the research and screening process. They first analyzed titles and abstracts. At the second stage, complete articles were selected for careful reading and were analyzed according to the eligibility criteria (inclusion/exclusion) for future data extraction. The concordance of the search results between the two reviewers was evaluated by Cohen’s Kappa (k) statistical test. Divergence among the reviewers was resolved through careful discussion, with the final decision being made by one author (EB). The authors of the included studies, when necessary, were contacted by e-mail to answer any questions that may have arisen.

Data Extraction and Analysis

Two reviewers (KV and ST) independently performed data extraction by reading the articles in their entirety. First author, year of study, study design, participant characteristics (number, age, gender, ethnicity, dentate or edentulous, and presence or absence of periodontitis), Mini Mental State Exam (MMSE) scores, magnetic resonance imaging (MRI), genetic risk (APOE-ε), periodontal clinical parameters (bleeding on probing (BOP), plaque index (PI), probing depth (PD), and clinical attachment level (CAL)), biomarker outcomes (IL-1β, IL-6, IL-10, and TNF-α), and IgG antibody for at least one periodontopathic bacteria. Due to the need to obtain relevant data, some authors were contacted for clarification of their study designs.

Two independent authors (BP and EB) conducted the quality assessment and evaluated the risk of bias in the included studies using the Risk Of Bias In Non-randomized Studies - of Exposures (ROBINS-E) tool [8]. This instrument is specifically designed to assess the internal validity of non-randomized studies of exposures, making it well-suited for this review. Its structured framework enables a systematic evaluation of potential biases arising from confounding, participant selection, exposure measurement, and outcome assessment. Since the studies included in this review primarily investigated the association between periodontal pathogens and/or related inflammatory biomarkers and AD, the ROBINS-E tool provided a robust, transparent approach to evaluating the methodological quality and reliability of the evidence.

Results

Study Selection

**Figure 1:**
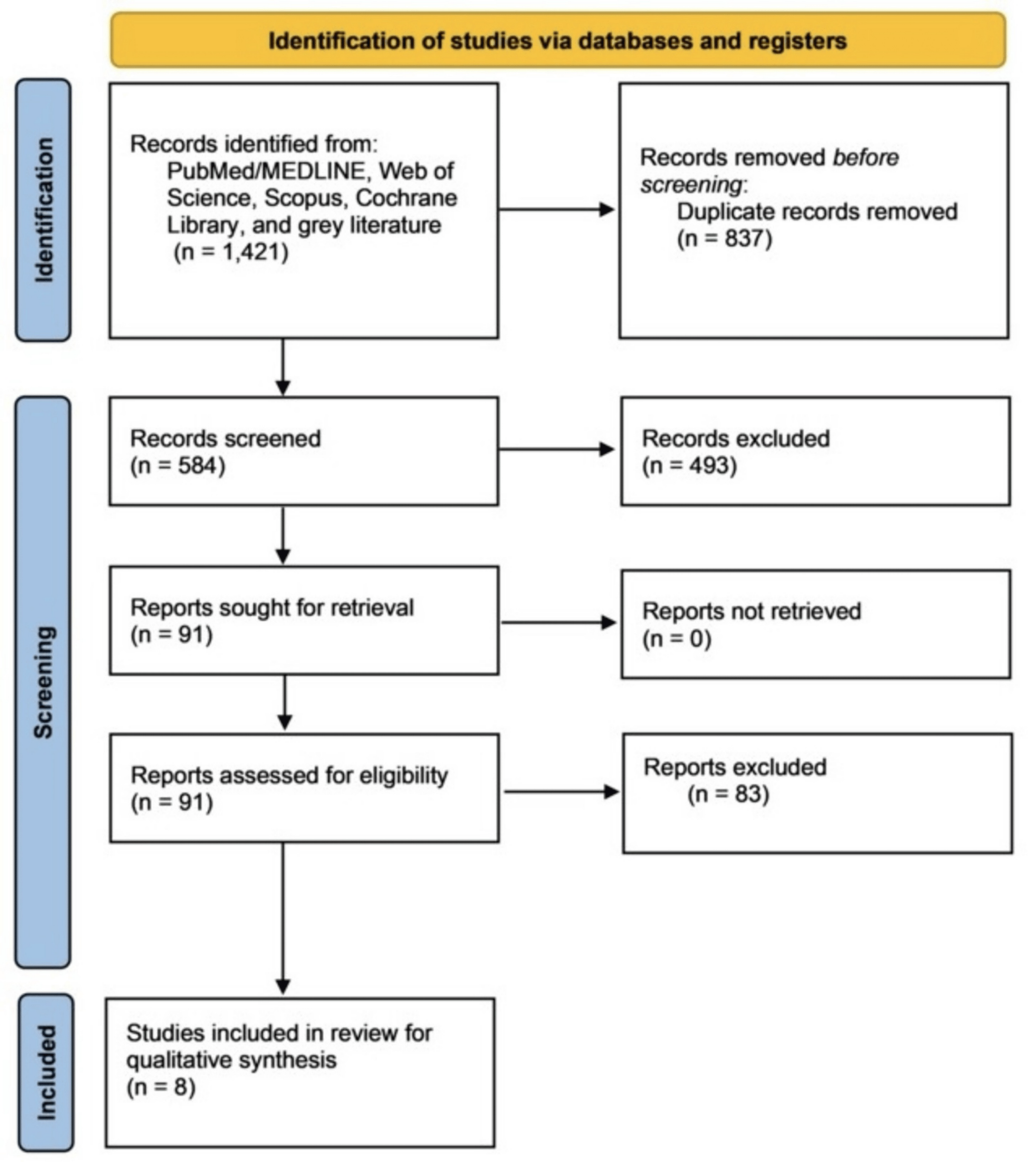
PRISMA flowchart. PRISMA: Preferred Reporting Items for Systematic Reviews and Meta-Analyses; MEDLINE: Medical Literature Analysis and Retrieval System Online

A total of 1,421 citations were obtained, comprising 593 studies from the PubMed database, 498 from the Web of Science database, and 330 from the Scopus database. After excluding duplicate studies, 584 studies remained for evaluation. Of these, 493 articles were excluded for not meeting the inclusion criteria. Ninety-one articles were read in full, and eight articles that fulfilled the inclusion criteria were included in the analysis. The k-value agreement between the two reviewers was 100% for both the potential articles to be included (based on titles and abstracts) and the selected articles. It was not possible to perform a meta-analysis due to the heterogeneous methodologies and data across studies. The selection process is described in Figure 1.

Risk of Bias Assessment

The risk of bias in the study methodology was evaluated using the ROBINS-E tool [8]. Of the eight studies included [9-16], none presented a low risk of bias. Three studies revealed a serious risk of bias [9,10,15], while five studies exhibited a moderate risk of bias [11-4,16]. The risk of bias analysis using the tool ROBIS-E is summarized in Table 2.

**Table 2:** Risk of Bias Assessment (ROBINS-E). ROBINS-E: Risk Of Bias In Non-randomized Studies - of Exposures

Authors, Year	DOMAIN 1: Confounding	DOMAIN 2: Selection of participants	DOMAIN 3: Classification of exposures	DOMAIN 4: Deviations from intended exposures	DOMAIN 5: Missing data	DOMAIN 6: Measurement of outcomes	DOMAIN 7: Selection of the reported result	Overall Risk of Bias
Kamer et al., 2009 [9]	Serious	Moderate	Moderate	Low	Moderate	Moderate	Moderate to serious	Serious
Stein et al., 2012 [10]	Moderate	Serious	Low	Moderate	Moderate	Moderate	Moderate	Serious
Noble et al., 2014 [11]	Moderate	Moderate	Moderate	Low	Moderate	Moderate	Moderate	Moderate
Cestari et al., 2016 [12]	Moderate	Moderate	Moderate	Low	Moderate	Moderate	Moderate	Moderate
Ide et al., 2016 [13]	Moderate	Moderate	Moderate	Low	Moderate	Moderate	Moderate	Moderate
Chen et al., 2017 [14]	Moderate	Moderate	Moderate	Low	Moderate	Moderate	Low	Moderate
Sochocka et al., 2017 [15]	Serious	Moderate	Moderate	Low	Moderate	Moderate	Moderate	Serious
Rubinstein et al., 2024 [16]	Moderate	Moderate	Moderate	Low	Moderate	Low	Moderate	Moderate

Characteristics of Included Studies

The characteristics of the selected studies are presented in Table 3. Eight studies fulfilled the inclusion criteria and were evaluated in this systematic review [9-16]. The number of participants in the included studies varied from 34 [9] to 219 [11], and their ages ranged from 40 [9] to 90 [14] years.

**Table 3:** Characteristics of the included studies. AD: Alzheimer’s disease; MCI: mild cognitive impairment; ADRD: Alzheimer’s disease and related dementia; Control: no AD; PP: periodontitis present; PA: periodontitis absent; NR: not reported; NO: not observed; CAL: clinical attachment level; PD: probing depth; BOP: bleeding on probing; PI: plaque index; CRP: C-reactive protein; NE: not explicit; WMH: white matter hyperintensity; SE: standard error; IQR: inter quartile range; MMSE: Mini Mental State Exam

Author(s), year	Study Design	Participant Characteristics	MMSE Results	MRI Results	Genetiс Risk (APOE-ε)	Periodontal Clinical Parameters	Biomarkers Outcomes	IgG Antibody	Follow-up	Conсlusions
Kamer et al, 2009 [9]	Case-control	N= 34, AD: 18 Control: 16, Gender: NR, Mean age: ~77 Mixed dentate/edentulous Periodontitis: 100% in both groups	AD: 19.5 (5.2), Control: 29.3 (.77)	NR	APOE-ε4 frequency is higher in AD 4/4 = two Alleles 4/3 = One ε4 and one ε3 allele 4/2 = one ε4 and one ε2 allele 3/3 = two ε3 alleles	NR	IL-1β AD: 6.9±3.5 Control: 11.6±15.9 IL-6 AD: 8.1±11.4 Control: 30.2±55.0 TNF-α AD: 13.0±4.3 Control: 8.2±4.7 P<.01	Elevated antibodies to AA in AD, AD: 13 (72%) Control: 6 (38%) (p= .042)	NR	Biomarkers such as IL-1β, IL-6, TNF-α, and antibodies against periodontal bacteria were elevated in patients with AD and are independently associated with AD, thus contributing to its diagnosis.
Stein et al., 2012 [10]	Cohort	N= 158, AD: 35, Male/Female: 9/26, Mean Age: 74.1 (7.5), MCI: 46, Male/Female: 24/22, Mean Age: 72.1 (6.1), Control: 77, Male/Female: 32/45, Mean Age: 70.0 (6.5), Periodontitis: 100% in all groups	AD: 28.8 (1.2) MCI: 28.8 (1.3) Control: 29.4 (0.8)	NR	APOE ε4 AD: 37.1% MCI: 37% Control: 15.6%	PD/CAL/BOP	NR	Elevated antibodies to* P. gingivalis, Aa, C. rectus, T. denticola, F. nucleatum, P. intermedia, and T.* forsythia in AD patients. *P. gingivalis*: (p=.007), Aa: NR *C. rectus*: NR, *T. denticola:* (p=.050), *F. nucleatum:* (p<.001), *P. intermedia:* (p<.001), *T Forsythia:* NR	18 years	There is an elevated host response to oral pathogens associated with periodontitis in patients with AD.
Noble et al., 2014 [11]	Case-cohort	N=219, AD: 110, Male/Female: 35/75, Mean Age: 78.9 (7.2), Control: 109, Male/Female: 36/73, Mean Age: 72.3 (4.6), Periodontitis: 100% in both groups	NR	NR	APOE-ε4 AD: 26 (24.1) Control: 28 (26.1)	NR	NR	Elevated antibodies to *P. gingivalis, *Aa, *C. rectus, T. denticola, A. naeslundii, E. nodatum T. forsythia* in AD patients: *P. gingivalis*: 25 (22.75%) Aa: 15 (13.6%) *C. rectus*: 72 (65.5%) *T. denticola:* 54 (49.1%) *A. naeslundii: *12 (10.9%) *E. nodatum*: 19 (17.3%) *T. forsythia:* 65 (59.1%) Non-AD patients: *P. gingivalis:* 25 (22.9%) Aa: 25 (9.2%) *C. rectus*: 78 (71.6%) *T. denticola: *62 (56.9%) *A. naeslundii: *10 (9.2%) *E. nodatum:* 23 (21.1%) *T. forsythia:* 72 (66.1%)	5 years	Serum IgG levels corresponding to common periodontal microbes were associated with an increased risk of developing AD.
Cestari et al., 2016 [12]	Cross-sectional case-control	N= 65, AD: 25, Male/Female: 10/15, Age Range: 63-92, Periodontitis: 40%, MCI: 19, Male/Female: 6/13, Age Range: 56–86, Control: 21, Male/Female: 7/14, Age Range: 62-87, Periodontitis: 42.8%	AD: 21.3 MCI: 27.6 ± 1.06 Control: 27.8±1.54 p<0.001	NR	NR	AD: PD: 4.68±2.15 CAL: 5.10±4.45 PI: 71.87±26.58 Control: PD: 4.52±2.32 CAL: 6.61±3.22 PI: 58.47±26.52	IL-1β AD: NO MCI: NO Control: NO IL-6 Higher levels in AD compared to controls, p=0.029 TNF-α Higher levels in AD compared to controls, p=0.005	NR	NR	There is a positive correlation between cytokine expression in the serum of patients with AD.
Ide et al., 2016 [13]	Cohort	N= 60 -1 PP: 22, Male/Female: 13/9, Mean Age: 74.9 (2.0), Control (PA): 37, Male/Female 17/20, Mean Age: 79.4 (1.3), Periodontitis: 100% in both groups	PP: 19.5 (1.0) Control (PA): 21.0 (0.9)	NR	NR	PD PP/ BOP	CRP AD (PP): .29 IQR (-.14, 1.44 µg/mL) Control (PA): -.02 IQR (-.72,.33 µg/mL) p=.08 IL-10 AD (PP): -.09 IQR (-.20,.06), pg/ml Control (PA): .05 IQR (-.05,.12 pg/ml). p=.047 TNF- α AD (PP): 02 SE (.12) pg/ml p=.03 Control (PA): .16 SE (.11), pg/ml p=.42	Elevated antibodies to* P. gingivalis* in AD. AD (PP): .38 (.02) Mean Difference= .01 (-.05 to .08), p=.70	6 months	Periodontitis is correlated with an increase in cognitive decline in AD, regardless of basal cognitive impairment status.
Chen et al., 2017 [14]	Matched- cohort	N= 27963, PP: 9291, Male/Female: 4940/4351, Periodontitis: 100% Control: 18,672, Male/Female: 9904/8768, Periodontitis: 0%	NR	NR	NR	NR	NR	NR	Up to 16 years	Findings demonstrated that 10-year CP exposure was associated with a 1.707-fold increase in the risk of developing AD. These results call for increased awareness due to the strong correlation.
Sochocka et al., 2017 [15]	Cross-sectional	N= 128 (all AD), Male/female: 45/83, Periodontitis: 100%, Control: None	AD: NE Median: 22	NR	NR	PD CAL PI BOP: median 51.2	IL-1β: 369 pg/ml LPS-Induced IL-1β: 2008pg/ml IL-6: 4693pg/ml LPS-induced IL-6: 31077pg/ml IL-10: 67pg/ml LPS-induced IL-10: 1314pg/ml TNF-α: 962pg/ml Median TNF-α: 62pg/ml LPS-induced	NR	NR	Periodontal disease, along with AD, shows comorbidity that may result in enhanced cognitive impairment.
Rubinstein et al., 2024 [16]	Cross-sectional	N= 486, Dementia: 8, MCI: 70, Control: 407, Mean age 74, Diverse ethnicity Dentate only	NR	MRI: Presence of cerebral microbleeds, presence of brain infarcts, white matter hyperintensity (WMH) volume, hippocampal volume, entorhinal cortex volume “AD signature” cortical thickness composite (reflecting AD-related neurodegeneration)	APOE-ε4 Present in 152 (31.3%) out of 486	PD ≥ 4 mm and CAL ≥ 4 mm in all teeth. Numbers not well defined	NR	Higher serum IgG to *P. gingivalis* was associated with higher entorhinal cortex volume. Higher serum IgG levels against *P. intermedia* were associated with greater hippocampal volume. Higher serum IgG levels against *F. nucleatum* were associated with a higher odds of cerebral microbleeds and infarcts.	≤ 4.5 years	In an elderly cohort, clinical, microbiological, and serological features of periodontitis were associated with MRI findings related to ADRD risk. In addition, CAL ≥4 mm has been associated with unfavorable MRI markers.

Cognitive Assessment

The cognitive deficits of participants in the five studies were evaluated using the MMSE [9,10,12,13,15]. In the study by Cestari et al., the scale was represented using a cut-off point (17/27) for the test group [12]. However, Noble et al. did not need to use any cognitive diagnosis method because the study participants were recruited from a project involving patients with dementia [11]. One study evaluated MRI markers associated with AD and related dementias (ADRD) [16]. The findings demonstrated that the clinical, microbiological, and host-response characteristics of periodontitis were associated with MRI indicators of increased risk of ADRD.

The APOE ε4 allele was used in four studies as a diagnostic criterion for AD [9-11,16]. The other studies used cognition questionnaires.

Periodontal Status Assessment

All the included studies evaluated the health of periodontal tissues during AD. However, only seven studies involved participants diagnosed with periodontitis [9-15]. Cestari et al. study involved participants with either healthy periodontal tissue (24%) or periodontitis (40%) [12]. In five studies, the periodontal clinical parameters were evaluated to diagnose periodontitis [10,12,13,15,16]. These studies correlated the clinical progression of periodontitis and AD with increased levels of pro-inflammatory biomarkers.

Inflammatory Biomarkers

Four studies evaluated inflammatory biomarkers (IL-6, IL-1β, IL-1, IL-10) and correlated their presence in periodontitis with AD progression [9,12,13,15]. These studies also assessed TNF-α. Three studies evaluated only IL-1β and IL-6 [9,12,15], while two studies analyzed only IL-10 [13,15]. In addition, one study analyzed C-reactive protein levels to assess the association between periodontitis and cognitive decline in AD, using serum levels of pro- and anti-inflammatory markers [13]. The most relevant results showed relatively increased C-reactive protein and TNF-α levels and decreased IL-10 in participants with AD and periodontitis during the follow-up period [9,12,15].

Antibodies Against Periodontal Pathogens

Among the studies included, five evaluated elevated IgG antibody levels against periodontal pathogens [9-11,13,16]. Noble et al. highlighted the presence of* Actinomyces naeslundii, Eubacterium nodatum, P. gingivalis, T. forsythia, T. denticola, Campylobacter rectus,* and *A. actinomycetemcomitans *in the test group (AD) [11]. High levels of *A. naeslundii *(HR53.1, CI 95%: 1.5-6.4) were associated with increased risk of AD progression. However, high levels of *E. nodatum* (HR 50.5, CI 95%: .2-.9) represented a lower risk for AD progression. In the study by Kamer et al., the parameter considered was the presence of at least one of the common periodontal pathogenic bacteria (*A. actinomycetemcomitans, P. gingivalis*, and *T. forsythia*) [9]. The number of antibodies against periodontal bacteria in AD patients (72%) was higher than in the control group without AD (38%). Furthermore, Ide et al. evaluated only the IgG of *P. gingivalis* and found no significant relationship between serum antibody levels for *P. gingivalis *and the rates of cognitive decline [13]. Another study demonstrated that an increased host response to oral pathogens (*P. gingivalis, T. denticola, C. rectus, Fusobacterium nucleatum*, and *P. Intermedia*) was associated with periodontitis in AD patients [10].

Discussion

The objective of this review was to investigate the relationship between periodontitis and AD progression by evaluating the presence of periodontal pathogens and pro- and anti-inflammatory biomarkers. All studies included systematically defined their inclusion and exclusion criteria. Another relevant aspect was the evaluation of the quality of the studies' sampling criteria. Five studies were rated as having a moderate risk of bias, while three were rated as having a serious risk of bias.

The MMSE is a commonly used test for assessing cognitive function in older people. This test was conducted across five of the included studies. The study by Sochocka et al. correlated MMSE scores and cognition with periodontal impairment and concluded that the greater the BOP, the greater the periodontal impairment and the lower the MMSE score, suggesting greater cognitive impairment [15]. The findings of Kamer et al. [9] corroborate those of Sochocka et al. [15] regarding the correlation between the MMSE score and AD in the presence of periodontitis. On the other hand, Ide et al. used the MMSE score solely as a secondary cognitive measure and reported no relevant results [13].

Cestari et al. noted that patients with AD exhibited significantly greater cognitive and functional impairment than both the mild cognitive impairment (MCI) and control groups, as reflected by lower MMSE scores and higher Pfeffer Functional Activities Questionnaire (FAQ) scores (p < 0.001) [12]. Despite this clear clinical difference, the authors found no statistically significant differences across the groups in objective periodontal measures, including mean and maximum probing pocket depth (PPD), CAL, O’Leary PI, and bleeding index, in their cross‑sectional comparison, indicating that while AD patients were more cognitively and functionally impaired, these particular periodontal indices did not differ between groups in this sample.

The presence of the ε4 allele of the APOE gene, established as the most common genetic risk factor for late-onset AD, was another evaluation criterion used in four studies [9-11,16]. However, the studies by Stein et al. [10] and Kamer et al. [9] used APOE protein status to assess AD status and MMSE scores to evaluate cognitive impairment in participants. The study by Noble et al. only used APOE in the presence of periodontitis, associating dementia with systemic inflammation [11]. The authors concluded that periodontitis could increase the risk of cognitive changes and subclinical brain damage. People with one or two APOE ε4 alleles are considered AD-positive. It must be emphasized that this factor is not enough to cause AD. This suggests that genetic and environmental factors, such as inflammation associated with periodontitis, may influence the progression of AD [11]. Kamer et al. [9] and Noble et al. [11] found that the presence of periodontal pathogens and the ε4 alleles of the APOE gene were correlated with cognitive decline in the presence of periodontal inflammation.

Another important aspect to consider in the study by Stein et al. is that the host's heightened response to oral pathogens associated with periodontitis may be elevated in patients even before neurological changes of AD are evident [10]. These results corroborate the findings of Noble et al., who showed that increased IgG levels in the presence of periodontal disease are associated with an incipient AD increase [11]. The longitudinal study by Stein et al. also addresses the temporality of the association between PD and AD as opposed to the other included studies that suggest only the association between high levels of antibodies for periodontitis and cognitive changes in a short follow-up period, and cannot establish the temporal nature of the association between the two diseases [10].

The inflammatory process is fundamental to the development of AD, and the presence of inflammation during AD supports the hypothesis that periodontitis can accelerate AD progression [2]. The studies included in this review showed that the release of large amounts of inflammatory biomarkers by periodontal tissues during periodontitis might play a central role in neuroinflammation [9,12,13,15]. These results corroborate the findings in other studies [17,18]. However, Noble et al. [11] and Stein et al. [10] did not find an association between AD progression and periodontitis-related inflammatory markers. Only one study included in this review evaluated C-reactive protein to investigate the association between periodontitis and cognitive decline during AD [13]. The results showed a relatively increased C-reactive protein level in participants during the follow-up period.

Oral bacteria have been detected in human brain tissue [19], and it has been proposed that these microorganisms may reach the brain and contribute to chronic infection and neuroinflammation. TNF-α and IL-6 levels were elevated in the blood of patients with AD. This study suggests their role in overlapping mechanisms linking oral infections and AD [12]. It has also been observed that TNF α, IL 1, and IL 6 can stimulate the synthesis of amyloid β 1-42 (Aβ₁₋₄₂) and the phosphorylation of tau protein (p-Tau), while Aβ₁₋₄₂ and p-Tau can further stimulate glial cells to produce TNF α, IL 1, and IL 6 [20]. This creates a positive feedback loop where chronic inflammation acts as both an initial trigger and a continuous accelerator of AD’s pathology.

A study found that both MCI and AD patients had similar plasma levels of TNF-α, which were significantly elevated compared to the levels of non-demented, healthy control subjects [21]. The studies included in the present review demonstrated that high levels of TNF-α during periodontal infections are correlated with increased cognitive decline and AD progression [9,12,13,15]. This finding aligns with other studies that have linked elevated TNF-α levels in individuals with dementia to the presence of periodontal pathogens and corresponding antibody responses [22,23].

Serum antibody levels for periodontal pathogens are significant markers for periodontal disease. Several studies have shown that patients with AD and periodontitis have elevated antibody levels against periodontal bacteria and increased plasma TNF-α levels [18,23]. These findings are similar to those of five studies included in this review, which showed that AD patients have a high antibody response to periodontal bacteria [9-22,13,16]. The most relevant periodontal pathogens found in AD are *A. naeslundii, P. gingivalis, T. forsythia, T. denticola, C. rectus, A. actinomycetemcomitans, F. nucleatum*, and *Prevotella intermedia*. However, Ide et al. did not find a positive association between serum antibody levels for *P. gingivalis* and cognitive decline rates [13]. The main result of the study by Noble et al. was that serum IgG levels are elevated when *A. naeslundii *is present and are associated with an increased risk of incident AD [11]. Additionally, a more careful discussion of the difficulties in using antibody levels as the exposure of interest should be included. The present review findings do not suggest that AD progression can be attributed solely to the presence of specific oral pathogens. Rather, the observed associations, including the increased risk associated with higher levels of* A. naeslundii*, should be interpreted within the broader context of age-related immune dysregulation. Antibody levels reflect exposure to pathogens and the host response, which can complicate interpretation in the setting of a chronic infection with multiple putative causal organisms. For this reason, antibodies against oral bacteria were interpreted in our study as markers of cumulative microbial exposure and host immune response rather than as disease-specific indicators. 

None of the studies reviewed performed periodontal treatment in AD patients. However, a recent study evaluated the effect of periodontal therapy on the preclinical stage of AD. Imaging markers of brain atrophy in late-onset AD and brain aging were used to assess whether periodontal treatment was associated with decreased brain atrophy and brain aging in these patients. The authors concluded that periodontal treatment delays brain atrophy but is not associated with a difference in brain age [24].

This systematic review has several limitations that should be acknowledged. Owing to the heterogeneity in study methodologies and designs, conducting a meta-analysis was not feasible. Additionally, the overall risk of bias among the included studies was moderate, with three studies exhibiting a high risk of bias. Therefore, the findings should be interpreted with caution. Future research on the prevention and treatment of periodontal disease in patients with AD should also explore whether a causal relationship exists between periodontal disease and AD. 

## Conclusions

Based on the studies included in this systematic review, inflammatory mediators associated with periodontitis are frequently observed alongside markers of neurodegeneration in AD. Elevated TNF-α levels and higher serum IgG antibodies to periodontal pathogens have been reported in some studies in individuals with cognitive decline; however, these associations do not establish a causal relationship. The findings suggest a potential link between chronic periodontal inflammation and neurodegenerative processes. Future prospective cohort and interventional studies are warranted to clarify the role of periodontal infection and inflammation in AD and to guide clinical strategies that may improve outcomes in AD populations.
